# Effectiveness of virtually delivered Body Project groups to prevent eating disorders in young women at risk: a protocol for a randomized controlled trial

**DOI:** 10.1186/s40337-023-00932-7

**Published:** 2023-11-24

**Authors:** Line Wisting, Eric Stice, Ata Ghaderi, Camilla Lindvall Dahlgren

**Affiliations:** 1 Regional Department for Eating Disorders, Division of Mental Health and Addiction, Oslo University Hospital, P.O. Box 4956, Nydalen, Oslo, 0424 Norway; 2 Department of Psychiatry and Behavioral Science, Stanford University, Stanford, USA; 3 Department of Clinical Neuroscience, Karolinska Institutet, Stockholm, Sweden; 4 Department of Psychology, Oslo New University College, Oslo, Norway

**Keywords:** Body image, Eating disorders, Prevention, Body project

## Abstract

**Background:**

Eating disorders (EDs) are a group of mental illnesses associated with significant psychological and physiological consequences. Overall, only about one-fifth of individuals with EDs receive treatment and treatment is effective for only about one-third for those who receive care. The development and implementation of effective prevention approaches for those at risk is therefore pivotal. The *Body Project* is the most effective ED prevention program for at-risk women according to several meta-analyses, but reach is limited since delivery, traditionally, has been in-person. Moreover, peer-led *Body Project* groups have been reported to produce stronger effects than clinician-led *Body Project* groups when delivered in-person. This has not yet been examined for virtually delivered *Body Project* groups. This study therefore seeks to investigate the effect of virtual *Body Project* groups delivered by peers versus clinicians on ED risk factors, ED symptoms, and prospective ED onset.

**Methods:**

Young women with body image concerns aged 16–25 years (N = 441) will be included in the study and randomized to three conditions: (i) virtually delivered *Body Project* groups led by clinicians; (ii) virtually delivered *Body Project* groups led by peers; and (iii) psychoeducational control group. Participants will complete assessments at five timepoints over two years (pretest, posttest, 6-months, 1-year, and 2-years).

**Discussion:**

Further research is needed to examine approaches to increase the potential for broad implementation of prevention of EDs. The virtual modality of the *Body Project* could markedly expand the reach for young women at risk. If findings confirm that peers can deliver virtual *Body Project* groups as effectively as clinicians, this will further enhance the implementation potential.

*Trial registration*: The present study has been registered on clinicaltrials.gov (NCT05993728).

## Background

EDs affect approximately 13% of women and 5% of males [1, 2], and are marked by chronicity and high rates of relapse, as well as substantial impairment, academic difficulties, depression, anxiety, self-harm, suicide, and morbidity [3, 4]. Risk factors for the development of EDs include female gender, adolescent age, internalization of the thin beauty ideal, body dissatisfaction, dietary restraint, and negative affect [5]. In Norway, results from a recent prevalence study in adolescents revealed that ED psychopathology was common (20%), and that the estimated prevalence of any ED was as high as 16% among adolescent girls [6]. These rates are comparable to those reported in other countries [7]. Yet, less than 20% of affected individuals receive treatment [8]. This underscores the importance of efforts to prevent the development of ED in high-risk populations. Selective ED prevention approaches specifically targets individuals at high-risk for EDs, i.e. young women > 15 years with body dissatisfaction [5].

The *Body Project*, a selective 4-hour group-based program designed specifically for young women at risk for EDs, has the strongest evidence of effectiveness in reducing ED risk factors (such as thin-ideal internalization and body dissatisfaction) and ED symptoms [9], as well as preventing future ED onset among young women [10]. The evidence base for the *Body Project* is strong and based on over 20 controlled trials including assessment-only controls [11–13] and alternative credible interventions [14, 15]. Participants who complete the *Body Project* have shown a 60% reduction in future onset of EDs relative to assessment-only control participants [14] and a 54–58% reduction in future onset of EDs relative to alternative interventions [15]. The *Body Project* was originally designed as a face-to-face intervention, limiting availability to those who could attend in person. To reduce barriers to access, Ghaderi et al. [16] examined the effectiveness of virtually delivered *Body Project* groups (1 h a week over four consecutive weeks), through Google Hangouts, among young women (N = 443) aged 15–20 years. The participants were randomized to three conditions; (i) virtually delivered *Body Project* groups; (ii) expressive writing; and (iii) wait-list control group. Groups were facilitated by psychology undergraduates. The wait-list group received the *Body Project* after the 6-month follow-up. At two-year follow-up, *Body Project* participants compared to control participants showed a 77% lower incidence of ED onset and significantly greater reductions in ED symptoms and risk factors. This suggests that virtual *Body Project* groups may be an effective prevention strategy for individuals at risk of EDs, with an increased outreach. Moreover, in-person *Body Project* groups led by peer group facilitators produced a 58% greater reduction in future onset of EDs over 4-year follow-up when implemented by peer educators versus clinicians [15], theoretically because peers are perceived as more similar to the participants and thus have greater credibility. Whether these findings extend to virtual *Body Project* groups is unclear. Peer-led groups demand fewer resources to carry out than clinician-led groups, which may facilitate broader implementation.

Finally, though the effect of the *Body Project* has a strong evidence-base, it is important to confirm that interventions affect objectively measured (as opposed to self-reported) outcomes. The *Body Project* has previously been found to affect four objective outcomes, including reductions in cardiac risk [17], brain reward region response to thin models [18], attentional bias for the thin ideal [19], and positive implicit attitudes toward the thin ideal [20]. However, no research has investigated whether the *Body Project* affects objective (implicit) outcomes when implemented virtually. More insight into such implicit and explicit mechanisms of change after *Body Project* participation may inform refinements of the script that will improve the effectiveness of the *Body Project*.

In sum, the *Body Project* has consistently demonstrated its effectiveness in reducing ED risk factors, symptoms, and future ED onset across several independent research teams and countries, including England [21], USA [15], China [22], Saudi Arabia [23], Mexico [24], and Brazil [25]. However, further steps are needed to increase reach, reduce practical barriers, and enhance the potential for broad implementation for individuals at risk. Further, the recently reported prevalence estimates of EDs in Norwegian youth [6] especially among girls, point to the need to confirm effectiveness of a brief evidence-based prevention program specifically targeting this at-risk group in Norway. Additionally, confirming whether peers can effectively deliver the *Body Project* in a virtual format [26] will be important for broad nation-wide implementation.

## Research aims and hypotheses

The overall objective of this study is to evaluate the effectiveness of the *Body Project*. Specifically, the following hypotheses will be tested:

1. Participants in both peer-led or clinician-led virtually delivered *Body Project* groups will show significantly greater reductions in ED risk factors and symptoms compared to controls.
2. Participants in both peer-led or clinician-led virtually delivered *Body Project* groups will show a significantly lower rates of ED onset over the 2-year follow-up than controls.
3. Participants in both peer-led or clinician-led virtually delivered *Body Project* groups will show significant reductions in positive implicit attitudes towards the thin ideal compared to controls.
4. Participants in peer-led (young female students) virtually delivered *Body Project* groups will show significantly greater improvements in outcomes than participants in clinician-led virtually delivered *Body Project* groups.

## Methods and design

### Design

This is a randomized controlled trial (RCT) with three arms; (i) clinician-led virtual *Body Project* groups; (ii) peer-led virtual *Body Project* groups; and (iii) psychoeducational control group.

## The intervention

The *Body Project* is a group-based ED prevention program based on the principles of cognitive dissonance. According to Festinger [27], cognitive dissonance occurs when there is an inconsistency between one’s beliefs and one’s actions. This inconsistency creates psychological discomfort, which motivates the individual to reduce cognitive discord by changing their beliefs to align with their actions. In the *Body Project*, participants collectively critique the thin beauty ideal through a series of verbal, written, and behavioral exercises. Engaging in these activities prompts participants to align their attitudes with their publicly displayed behaviors, which results in a reduction in pursuit of the thin beauty ideal, which in turn is theorized to reduce body dissatisfaction and ED symptoms. Consistent with this theory, reductions in pursuit of the thin beauty ideal mediated the effects of the *Body Project* on ED symptom reduction [28]. During the four sessions, the participants define the appearance ideal, discuss costs of pursuing this ideal, attempt to dissuade group facilitators from pursuing the appearance ideal in role-plays, and engage in discussions about the negative effects of social comparison [29] and social media. Written home exercises are conducted between sessions and debriefed with the group in the following meeting. We have previously translated the latest version of the *Body Project* script [15] to Norwegian for the first time and piloted a diabetes-specific version among Norwegian young women with type 1 diabetes [30]. No further adaptations were considered necessary to obtain cultural fit, which is similar to experiences reported in the Swedish virtual *Body Project* study [16]. The *Body Project* is largely participant-driven given the Socratic open questions in line with dissonance-theory, which may explain why minimal changes are required to obtain efficacy in different countries. For example, in session 1 group facilitators ask what the society tells us that young women should ideally look like, by which the participants themselves define the appearance ideal in their own cultural context.

### Participants

A total of 441 young women will be included in the study. Inclusion criteria are being female, age between 16 and 25 years, and at least some level of self-reported body dissatisfaction verbally confirmed by the participant in the screening call, following procedures used in previous trials [14, 15, 31]. Participants diagnosed with an ED will also be included. These individuals have shown to display larger reductions in ED symptoms than participants without EDs in response to the *Body Project d* = 0.71 vs. 0.17) [32]. Exclusion criteria include being hospitalized or currently in treatment for an ED.

### Procedure

A Norwegian website (www.bodyproject.no) has been designed to facilitate recruitment and project profiles are posted on Facebook and Instagram. The link will be shared via our online platforms, including Facebook, Instagram, and TikTok. Also, advertisement campaigns on social media will be run nationally. Individuals express interest by sending an e-mail to a *Body Project* mailbox hosted by Oslo University Hospital, which leads to a screening phone call to determine eligibility based on the inclusion criteria described above. In this screening call eligible participants verbally confirm to experience at least some body dissatisfaction. After providing informed consent, participants will complete baseline assessment (pretest) before being randomized into one of three arms: (i) peer-led virtual *Body Project* groups; (ii) clinician-led virtual *Body Project* groups; and (iii) educational video control group. Assessment will be repeated post intervention (posttest) and follow-up assessment after 6-months, 1-year, and 2-years. ED diagnostic interviews are conducted at baseline, 1-year, and 2-year assessments by research staff who are masked to the condition to which each participant will be randomized. Online assessment will be conducted via an online survey (Viedoc) hosted by Clinical Trials Unit (CTU), Oslo University Hospital. Randomization will be conducted by Viedoc once baseline assessment is completed. Virtually delivered *Body Project* group sessions will be implemented by use of Microsoft Teams, administered by Oslo University Hospital. Adaptations required for virtual script delivery include administration of home exercises via e-mail and using the chat function for summarizing themes during in-session group discussions rather than white boards or flip-charts used for in-person *Body Project* groups. This study is funded by the Norwegian Health Authority Region South-East (reference 2,023,032).

### Group facilitators

Clinician group facilitators consist of nurses (psychiatric and school health nurses), clinical dietitians, and a social worker, who are licensed health care personnel associated with the Regional Department for Eating Disorders at Oslo Universal Hospital. Peer group facilitators are recruited through acquaintances of our research team, and includes university and college-level students (not within medicine, psychology, or nursing) and maximum four years older than the participants.

### Training and supervision

Facilitators (both clinicians and peer educators) will read the intervention manual that outlines the theoretical rational of the intervention, reviews the evidence-base for the *Body Project*, and contains a detailed intervention script. Facilitators will then attend an 8-hour virtual workshop, where the conceptual rationale and supporting evidence for the *Body Project* are presented, facilitators role-play delivery of the intervention using the scripted manual, and process issues are discussed (e.g., promoting session attendance and homework completion, and recording attendance, homework completion, and verbal participation in the sessions for each group participant). All *Body Project* sessions will be videotaped, and all sessions from the first group implemented by facilitators will be rated for intervention fidelity and therapeutic competence (see below for psychometrics); supervision based on these ratings will be provided to facilitators via e-mail (feedback on what went well, and advice on how certain aspects may be improved for future sessions), which has been found to significantly increase intervention effects compared to trials that did not provide this type of ongoing supervision [10]. This study will use the same procedures for training and supervision as past *Body Project* trials [31, 33].

#### Control condition

An educational control condition was selected to control for expectancy effects and demand characteristics. Pre-existing freely available videos allow participants to view them over the Internet to match the virtual delivery modality (1 h per week over 4 consecutive weeks). The videos contain information about how to promote a positive body image, costs associated with EDs, and how to seek help if they experience concerns related to body image, eating, body pressures, and weight. Past trials of the *Body Project* have utilized such educational videos as the control condition [15, 33, 34]. Results indicated that participants assigned to the educational control condition showed reductions in ED symptoms compared to assessment-only controls [34], but that the reductions in ED symptoms were significantly larger for *Body Project* participants than for control participants. Adherence to watching the videos will be recorded by self-report at posttest assessment. In a previous trial participants showed similar adherence to attending *Body Project* group sessions versus watching the educational videos (typically over 80%) [35].

### Assessment

All measures are translated to Norwegian. Only the Eating Disorder Examination Questionnaire (EDE-Q) and Eating Disorder Assessment for DSM-5 (EDA-5) are validated in Norwegian, while the psychometric properties of the remaining measures are validated in English. See details below:

*Thin-ideal internalization*: the 8-item Ideal-Body Stereotype Scale-Revised (IBSS-R) [36] to assess endorsement of thin-ideal using a Likert scale ranging from 1 = strongly disagree to 5 = strongly agree. It has shown internal consistency (α = 0.91), 2-week test-retest reliability (*r* = .80), predictive validity for future ED onset, and sensitivity to detecting intervention effects [33].

*Body dissatisfaction*: ten items from the Body Parts Scale [37] to assess dissatisfaction with body parts with a Likert scale ranging from 1 = extremely dissatisfied to 6 = extremely satisfied. It has shown internal consistency (α = 0.94), 3-week test-retest reliability (*r* = .90), predictive validity for future ED onset, and sensitivity to detecting intervention effects [33].

*Negative affect*: 20 negative items from the Positive and Negative Affect Schedule – Revised (PANAS-X) [38] to measure negative affect. It has high internal consistency (mean Cronbach’s α of 0.94), test-retest reliability (r = .78), and convergent validity [15].

*ED symptoms*: The Eating Disorder Examination Questionnaire (EDE-Q) [39] is a self-report 28-item questionnaire of specific eating disorder psychopathology based on the Eating Disorder Examination (EDE) diagnostic interview [40]. Reponses range from 0 to 6 with higher scores indicating higher levels of ED psychopathology. The EDE-Q consists of the four subscales *eating restraint*, *eating concern*, *shape concern*, and *weight concern* and is previously translated and validated in Norwegian men and women [41, 42].

*Appearance ideal internalization and pressures*: the Social Attitudes Towards Appearance Questionnaire 4 – Revised (SATAQ-4R) for women [43] consists of 31 items with subscales which measure: internalization thin/low body fat, internalization of muscularity, internalization of general attractiveness, pressures from family, pressures from peers, pressures from significant others, and pressures from media. Internal consistency (Cronbach’s α ≥ 0.82) and construct validity have been demonstrated.

*Implicit attitudes*: the Implicit Association Test (IAT) [44] measures implicit attitudes to the thin beauty ideal. The IAT is a widely used measure of implicit attitudes based on the strength of automatic associations between concepts and positive versus negative adjectives, which assesses attitudes toward images of the thin appearance ideal and words describing ED behaviors. It has shown good psychometric properties [45]. Participants are asked to quickly sort words into categories in a series of seven blocks that vary in whether the thin images and ED words are paired with positive or negative words. Response latencies are recorded according to standard.

*ED diagnoses*: the Eating Disorder Assessment for DSM-5 (EDA-5). The EDA-5 [46] is as a semi-structured, web-based diagnostic interview assessing DSM-5 feeding and eating disorders that have been present in the previous three months. The interview can be administered with limited training [47] and is significantly quicker to administer than traditional diagnostic interviews for EDs. High rates of agreement have been found between diagnoses by EDA-5 and traditional clinical interviews, such as the Eating Disorder Examination (EDE) interview, supporting its diagnostic validity [46, 48]. A recent validation study [48] supports the validity of the Norwegian EDA-5 to efficiently generate ED diagnoses without compromising diagnostic accuracy.

### Implementation fidelity and facilitator competence

All sessions will be recorded and key elements of a randomly selected subset of sessions will be rated for degree of fidelity (100-point scale from 0 = No adherence; the section was skipped to 100 = Perfect; all material in the section was presented as written). Facilitator competence will be rated with 12 items (e.g., leaders express ideas clearly and at an appropriate pace) using a 100-point scale with anchors for each item (e.g., 20 = Poor; leaders are difficult to follow and session proceeds at an uncomfortable pace, 100 = Superior; leaders are unusually articulate and express ideas in ways that all group members understand; perfect pace). Fidelity ratings and competence ratings have shown inter-rater agreement (ICC = 0.92 and = 0.96, respectively) for the original *Body Project* [49]. The PI will be responsible to do these ratings and provide supervision to group facilitators.

### Statistical analyses

*Model building.* Intent-to-treat (ITT) analyses of condition effects will be evaluated using mixed effects growth models fit with SAS 9.2 PROC MIXED [50]. Individual variability in outcomes from posttest to 2-year follow-up was modeled adjusting for pretest outcome values. Following Singer and Willet [51], when constructing the longitudinal models we will (a) examine empirical growth plots; (b) fit an unconditional means model; (c) fit an unconditional linear growth model; (d) fit unconditional non-linear models; and (e) compare models of longitudinal change using the Akaike Information Criterion. Condition, time (coded in months since posttest), and a condition × time interaction will be added to the unconditional model. We will estimate a growth model for each continuous outcome. We will report the results of planned contrasts from these models, contrasting each pair of conditions, reporting condition differences in the model implied least-square means derived from the time and condition × time interactions at 1- and 2-year follow-up. We will report effect sizes that are equivalent to Cohen’s *d* [52]. We will use full information maximum likelihood (ML) estimation to impute missing data because this intent-to-treat approach produces more accurate and efficient parameter estimates than alternative analytic approaches such as analyzing only participants who provide complete data, making multiple imputation the recommended procedure for handling missing data [53]. Missing data will be imputed using PROC MI using baseline levels of the outcomes, condition, and demographic factors (age, race, and parent education). Based on the recommendations [53], we will impute 50 data sets. Model parameters and standard errors were combined following Rubin [54] and implemented using PROC MIANALYZE. Cox proportional hazard models, fit with STATA [55] will test whether eating disorder onset over 2-year follow-up was significantly lower in each condition versus each other condition. Schoenfeld residuals will be examined to test the proportional hazards assumption. Hazard ratios and number needed to treat (NNT; [56]) are provided as measures of effect size. Because hazard models accommodate right censoring, we will not impute missing incidence data. Given that 88% of the effects from previous *Body Project* trials have replicated in 22 controlled trials from independent teams [15], we will use a p-value of 0.05 for all inferential tests because this usually balances risk for type I and type II errors.

### Power calculation

Sample size calculation was made for incidence of EDs assessed over a 2-year follow up via diagnostic interviews as the primary endpoint. Incidence rates were obtained from the previous Swedish virtual *Body Project* RCT [16]. Based on this trial we postulated a difference in incidence of EDs of 8.8% and 2% between groups, and a hazard ratio of 0.26 during 2 years of follow-up. Then, with a significance level of 5% and a power of 90% the estimated number of patients needed was 147 in each arm, the total number needed was 441.

## Discussion

### Ethical considerations

The study is registered in clinicaltrials.gov (NCT05993728) and approved by the data protection department at Oslo University Hospital (23/14,559) and the Regional Ethics Committee (609,734) in Norway. Informed consent will be gathered, and data will be handled and stored according to requirements. Potential ethical concerns include (i) participants could somehow be identified in the video-recorded sessions or research records might be obtained by someone not authorized to hear/view them. Although this could be moderately distressing due to the personal nature of the data, the likelihood of this risk is very low given our plans for protection against risks, in collaboration with the CTU at Oslo University Hospital who will help with safety and quality monitoring of the study. A data safety and monitoring board (DSMB) will be formed to provide monitoring on a regular basis. Participant data will be identified by their assigned ID number rather than by name, and efforts will be made to ensure participants’ full names are not used in the sessions; (ii) participants might discuss confidential information about another group member outside the group. This could be distressing but also appears unlikely based on our previous experience and our plans for protection (of the over 150 *Body Project* groups we have conducted, this problem has never come to our attention); (iii) participants may experience mental health deterioration that requires additional care; and (iv) participants may experience a dangerous ED-related medical risk. Our team is not providing any medical treatment or guidance, however participants are offered a phone call with a therapist for advice on local treatment options if necessary. Based on past experience with other studies in our team, a few participants have accepted this invitation, which has worked well.

### Publication plan

Research process and outcomes will be shared at national and international conferences, health nurses at high schools, user organizations, and on social media. Academic papers arising from this project will be published in international peer-review journals and working titles include: **a**) the effectiveness of virtually-delivered *Body Project* on ED risk factors and symptoms compared to controls; **b**) the effectiveness of virtually-delivered peer-led versus clinician-led *Body Project* groups on ED risk factors and symptoms; and **c**) incidence of EDs at 2-year follow-up among participants receiving virtual *Body Project* versus control condition.

### Potential impact

A documented effect of virtually delivered brief ED prevention program, which requires little time to train group-facilitators, may be important for health nurses at high-schools, and for their students, which represents the age group with the peak onset of EDs. Thus, this is a critical window for effective prevention. We would offer to freely share the manual and provide training to implement this in high schools. Moreover, if the proposed study finds that peers are as effective as clinicians in delivering virtual *Body Project* groups, this would increase the implementation potential even further. Thus, the opportunities for generalization and broad application of knowledge are considered significant with the proposed study. The *Body Project* is brief in nature and requires few resources to carry out, including training of group facilitators. The *Body Project* has already been successfully implemented by a range of different research groups in a variety of countries and settings and is even effective when implemented by peers/students, reducing implementation expenses [57]. The novel virtual delivery modality of the *Body Project* could markedly expand the reach of the ED prevention program for young women at risk, who often do not reside near clinics or other in-person *Body Project* sites.

### Future Body Project plans

The *Body Project* is a broader project at the Regional Department for Eating Disorders at Oslo University Hospital, consisting of ongoing and future sub studies within our project group. Current *Body Project* studies include: **(i)** *Diabetes Body Project;* the JDRF-funded multi-site RCT is recently initiated and will run broadly parallel processes with the current study; **(ii)** *Young Athlete Body Project;* a pilot study among young athletes at sports junior high schools, in collaboration with the Norwegian School of Sport Sciences (NIH) and Østfold University College; and (iii) *Body Project Treatment*, a novel 8-hour transdiagnostic outpatient treatment for individuals with an established DSM-5 ED diagnosis, which is an extension of the *Body Project* prevention program. Finally, we are in the process of seeking financial support to develop and evaluate a version of the *Body Project* specifically targeting young men.

## Data Availability

Not applicable.

## Abbreviations

EDs: Eating disorders
RCT: Randomized controlled trial
CTU: Clinical Trials Unit
IBSS-R: Ideal body stereotype – revised
DRES: Dutch restrained eating scale
PANAS-X: Positive and negative affect schedule
IAT: Implicit association test
ICC: Intraclass correlation
PI: Principle investigator
ANCOVA: Analyses of covariance
MI: Multiple imputation
MAR: Missing at random
